# Connectomics Annotation Metadata Standardization for Increased Accessibility and Queryability

**DOI:** 10.3389/fninf.2022.828458

**Published:** 2022-05-16

**Authors:** Morgan Sanchez, Dymon Moore, Erik C. Johnson, Brock Wester, Jeff W. Lichtman, William Gray-Roncal

**Affiliations:** ^1^Research and Exploratory Development Department, Johns Hopkins University Applied Physics Laboratory, Laurel, MD, United States; ^2^Department of Molecular and Cellular Biology, Harvard University, Cambridge, MA, United States

**Keywords:** connectome, annotation, software, standard, queries, reproducibility

## Abstract

Neuroscientists can leverage technological advances to image neural tissue across a range of different scales, potentially forming the basis for the next generation of brain atlases and circuit reconstructions at submicron resolution, using Electron Microscopy and X-ray Microtomography modalities. However, there is variability in data collection, annotation, and storage approaches, which limits effective comparative and secondary analysis. There has been great progress in standardizing interfaces for large-scale spatial image data, but more work is needed to standardize annotations, especially metadata associated with neuroanatomical entities. Standardization will enable validation, sharing, and replication, greatly amplifying investment throughout the connectomics community. We share key design considerations and a usecase developed for metadata for a recent large-scale dataset.

## 1. Introduction

In an effort to better understand structural organization and anatomy of nervous systems at nanoscale spatial resolution, increasingly large, even petascale, connectomics datasets have been collected using Electron Microscopy (EM) and X-Ray Microtomography (XRM) (Kasthuri et al., 2015; Schneider-Mizell et al., 2020; Xu et al., 2020; Consortium et al., 2021; Shapson-Coe et al., 2021; Witvliet et al., 2021). Currently, researchers and automated algorithms can label cells, subcellular components, and connections between cells to generate brain networks. Formats of such annotations, however, can vary greatly between datasets and institutions. As such, the computational expertise required to explore large, unfamiliar datasets and understand heterogeneous raw annotations remains a serious barrier to their widespread reuse, such as for downstream analysis of previously-collected and potentially unfamiliar data. Consequently, there is demand for simple community-adopted standards for storing key information about neuroanatomical entities represented in EM, XRM, and correlated light microscopy (LM) datasets as well as software tools built upon these standards to allow any researcher to quickly and easily extract information on annotated bodies without grappling with raw annotation downloads and lab-specific post-processing pipelines. This work focuses on filling a key need for the community by addressing a central aspect of annotation variability. It calls for standardized storage of metadata associated with key neuroanatomical entities, such as neurons, synapses, and organelles to supplement raw annotations. It also suggests an approach to metadata standardization through the use of community-adopted definitions, and demonstrates an example of how such standards can facilitate the development of simple data exploration interfaces.

Raw annotations may have any or all of the following formats: segmentations, anatomical skeletons and meshes, synaptic connectivity networks, and information in the form of tables or network attributes. From dataset to dataset, each of these primary data formats and associated documentation can vary in terms of structure, meaning, and transparency, making it difficult to use them to accurately extract relevant information about commonly-studied entities and test simple hypotheses that may not rely on spatial representations of the data. This motivates the use of standardized formats for storing annotated objects along with key attributes, separate from a lab's chosen raw annotation format, which may store such information indirectly. (i.e., The number of synapses on a neurite can be extracted through segmentation post-processing).

In this period of growth in EM, XRM, and correlated LM imaging, and their increased adoption and utilization in neuroscience, it would be advantageous to implement standards that ensure interoperability and sustainability, beyond just availability of these datasets through public release. This will promote rapid analysis, true openness, sharing between laboratories, and reproducible results in connectomics research. Existing annotation formats serve their purposes within labs, but extraction of neuroanatomical entities and properties in a standardized format can facilitate cross-institutional collaboration and exploration that existing formats do not always permit. Further, such standardization will enable the development of existing, as well as additional shared computational tools with user-friendly interfaces for querying these unique data for scientific discovery regardless of a user's computational expertise.

Because these datasets are large and complex, it is especially important to promote data exploration and discovery. Visualization and querying tools exist already, but are often lab- or dataset-specific (Clements et al., 2020). Furthermore, developers of new software must choose a data representation to support, which limits each new tool's broad applicability. One benefit of annotation standards is the potential for mitigation of this challenge through design and modification of software tools to build upon annotation standards. Visualization and querying software such as Neuroglancer (Maitin-Shepard, 2020), Neuromorpho (Ascoli et al., 2007), DotMotif (Matelsky et al., 2021), Webknossos (Boergens et al., 2017), NeuPrint (Clements et al., 2020), and others (Yatsenko et al., 2015) can be modified to support community-developed annotation standards and even integrated into a standards-supported, centralized discovery portal geared toward users without extensive computational backgrounds. Such a centralized connectomics discovery platform that allows exploration of datasets across imaging modalities, organisms, and institutions, is an exciting prospect, and is most feasible once metadata standardization is adopted.

This work will discuss the need for annotation metadata standards, propose a framework for such standardization, and demonstrate an application of such standards. To demonstrate potential impacts of standards on analysis software, we provide a case study in which we build tools to store and query a large emerging human connectome dataset, H01 (Shapson-Coe et al., 2021).

## 2. Annotation Standards

An acknowledged challenge in the field of connectomics is mitigating the impact of highly varied annotation representation on software and institution-level interoperability (Plaza et al., 2014). As the field grows and data volumes increase, the necessity for sharing data through remote and programmatic interfaces increases, and, in turn, the need for community-developed algorithms and software to extract and process that data also grows. Answering this challenge requires creating and popularizing annotation representation standards which enable parsing and understanding the scenes present in these nanoscale neuroimaging volumes, without alienating researchers with existing analysis pipelines.

Because the fields of EM and XRM data are still emerging, defining standards for these communities is timely. In order to enable community-oriented connectomics frameworks and collaboration, new annotation standards and software tools built to support those standards must strike a balance between organization and flexibility which is why we focus on standardized, expandable neuroanatomical entity definitions to store metadata as opposed to restricting raw annotation formats.

### 2.1. Support Common Raw Annotation Formats

The call for metadata standardization does not necessitate abandonment of existing raw annotation representation formats. Abandoning these formats could lead to obsolescence of existing, useful annotation, and analysis software (Ascoli et al., 2007; Saalfeld et al., 2009; Boergens et al., 2017; Berger et al., 2018) and ultimately alienation of institutions with incompatible formats, and is not the focus of this article. Instead, we hope to provide a blueprint for a new export format and urge institutions to build import/export tools. New standards, therefore, can continue to support a variety of common data representations.

Though not the focus of this article, the authors recognize that raw annotation formats could benefit from improvements as well, specifically in terms of documentation. Further work could better connect metadata to raw annotations and convey how neuroanatomical entities are represented in raw annotation data.

### 2.2. Facilitate Connections Between Datasets

Additionally, community-adopted annotation standards can enable linkage between data modalities and datasets. This facilitates comparison, meta-analysis, and registration with other datasets and imaging modalities. Links to different data modalities such as those between structural and functional LM data for the same subject, can encourage exciting research relating structure to function at the synaptic level (Consortium et al., 2021), and links between datasets can facilitate analysis across brain regions, individuals, and species, paving the way for understanding what is conserved and what differs across datasets and enabling large-scale discovery.

### 2.3. Metadata Standardization

Storage of metadata associated with neuroanatomical entities needs to be standardized to promote reproducibility, extensibility, and queryability of connectomics metadata. As such, new metadata standards must be built around the core knowledge products extracted from neurons, synapses, and their relationships (e.g., connectivity). Further, because user needs for data processing are diverse, standards must be conducive to common nanoscale connectomics research questions, such as those pertaining to location, topology, morphology, and cell types (LaGrow et al., 2018) as well as those surrounding connectivity at a local or circuit level (Matelsky et al., 2021) and even at higher-levels pertaining to brain regions and white matter tracts (Sporns et al., 2004; Bassett and Bullmore, 2006).

To satisfy diverse user needs as well as the need for standardization, we propose that the community agree upon definitions and minimum required attributes for key entities extracted from connectomics data, which are relevant to a variety of research areas. In Table 1, we define several areas and examples for annotation metadata standardization. For each neuroanatomical entity in the dataset (e.g., neuron, synapse, neurite, etc.) data owners should provide the URI, data representation, type, and, when applicable, links to other entities. Additionally, entities can be either user-defined or community-defined. Though data owners have the option to define new entities (e.g., user-defined), there are several entities which should have community-adopted definitions and properties. This combination of entities appropriately balances structure and flexibility in a way that allows software built upon standards to extract information uniformly across datasets while also allowing researchers to store additional non-standardized entities and metadata as desired. It also allows researchers to choose the level of granularity at which to store a dataset. Though community definitions will exist for multiple levels (e.g., from vesicles and mitochondria to neurons and layers), not every dataset needs to include all of these entities. Larger datasets, for example, may only include higher-level entities, while smaller datasets might contain lower-level entities. The only stipulation is that if a dataset chooses to include a particular entity, that entity's minimum properties must be satisfied.

**Table 1:** Key annotation metadata definitions.

Uniform resource identifier (URI)	A link to specify where source data is located
Links	URIs to parent, child, sibling relationships
Data representation	The format used to represent a neuroanatomical entity (e.g., skeletons, meshes, or pixels)
Entity	An object with neuroscience significance (e.g., RAMON types: neurons, synapses, organelles); has properties
Property	An attribute and value such as weight, cell type, or layer
Community-defined entity	An entity with a community-adopted definition and a minimum set of required properties; can be extended
Community-defined property	A property with a community-adopted definition
User-defined entity	An entity without a community-adopted definition or minimum set of required properties; an entity defined by the user
User-defined property	A property without a community-adopted definition; a property defined by the user

Our approach to annotation representation and metadata follows a neuroscience schema, previously used internally, called Reusable Annotation Markup for Open Neuroscience (RAMON) (Gray Roncal et al., 2015). RAMON defines a minimum set of annotation types and associated metadata that capture important biological information as well as relationships between annotations that are critical for connectome generation and neuroscientific exploration.

In particular, the H01 synapse annotation type includes metadata such as synapse id, type, and associated neurons. Currently, RAMON defines metadata standards for biological entities which are used commonly across connectomics datasets, such as neurons, synapses, and organelles, although this can be extended to additional entity types as needed.

## 3. Annotation Queries

As mentioned previously, one benefit of metadata standardization is that it enables the development of tools to query data, regardless of its origin. Through queries, researchers can characterize networks, extract patterns, and relate these patterns to function. Currently, however, asking even basic, fundamental questions (e.g., how many, how much) about a new dataset can be challenging from both a standardization and computational complexity perspective. Though previous work has presented information extraction tools for specific datasets and institutions (Clements et al., 2020), metadata standardization has the potential to expand the use of existing tools cross-institutionally, foster the development of new ones, and facilitate integration of numerous tools into a single location. The community would benefit from a shared discovery portal built upon community archives and standards (Ascoli et al., 2007; Sunkin et al., 2012; Vogelstein et al., 2018; Rübel et al., 2019), which provides broad accessibility to EM and XRM data and annotations through query submission tools to enable deeper understanding of these data. In this work, we demonstrate this particular benefit of metadata standardization through the development of a simple querying tool built upon RAMON.

Ideally, researchers should be able to easily query counts, distributions, properties, and connectivity of neuroanatomical entities as well as image and graph metrics for any connectomics dataset, regardless of source institution. Queries such as number of synapses in a given region, or the distribution of synapses on a particular neuron type could help answer a variety of research questions, but the broad community interested in brain atlases and neuroanatomy has traditionally had little access to and experience with large-scale EM and XRM datasets. Tools for executing standardized queries could, therefore, enable a new wave of discoveries.

## 4. Case Study: H01 Human Data

Here, we present a case study, in which we store annotations from a petascale human cortex dataset, the H01 dataset (Shapson-Coe et al., 2021) and build tools to that allow users to access and query that data through a web application. The H01 dataset consists of a cubic millimeter volume with annotations for 50,000 cells, hundreds of millions of neurites, and 150 million synapses, taken from a human surgical sample from the temporal lobe of the cerebral cortex. This dataset was chosen because of its size, breadth of annotation types, and significance as the first large, nanoscale human connectomics dataset. To demonstrate the robustness and generalizability of our approach, we also include a second dataset (Kasthuri et al., 2015) in our database and query engine.

### 4.1. Software Architecture

As a demonstration of the power of metadata standardization, and to shed light on neuroanatomy in the human cortex, we developed a connectomics query engine which supports the analysis of the H01 dataset. Our application, called the H01 Community Discovery Portal, is currently deployed in the Amazon Web Services cloud and follows the Representational State Transfer (REST) software architecture to ensure flexibility for storage of neuroanatomical metadata. The discovery portal consists of a Flask-based (Grinberg, 2018) web application, which serves as a user-friendly interface for researchers to explore and query the H01 dataset, a standards-supported H01 database, and a Flask-based Application Programming Interface (API) to enable easy access to the H01 dataset. We note that this is a simple example demonstrating the concepts outlined in this article and additional systems (e.g., neuPrint, CATMAID) might be used as robust alternatives with an appropriate schema.

The API is a web service consisting of eight RESTful web endpoints which retrieve and return H01 synapse, neuron, and layer data when a specific URL is accessed over Hypertext Transfer Protocol. The H01 Community Discovery Portal stores annotation metadata in a document-oriented, MongoDB (Chodorow, 2013) database.

### 4.2. Storing and Accessing the H01 Data

In the H01 database, we store nearly 27 GB of synapse, neuron, and layer properties along with their properties as MongoDB collections as described below:

- **Neuron Object:** Neuron ID (Integer), Volume (NumberLong), No. Outgoing Synapses (Integer), No. Incoming Synapses (Integer), No. Incoming Excitatory Synapses (Integer), No. Incoming Inhibitory Synapses (Integer), No. Dendrite Skeleton Nodes (Integer), No. Axon Skeleton Nodes (Integer), No. Dendritic Spines Skeleton Nodes (Integer), No. Cilia Skeleton Nodes (Integer), No. Axon Initial Segment Skeleton Nodes (Integer), No. Myelinated Axon Skeleton Nodes (Integer), Spinyness (Double), Layer (Integer), Neuron Type (Integer), Excitatory/Inhibitory Synapse Balance (Double)
- **Layer Object:** Layer Width (Integer)
- **Synapse Object:** Synapse ID (ObjectID), Synapse Type (Integer), Pre-synaptic site (Object), Post-synaptic partner (Object), Location (Integer), Bounding Box (Integer), Layer (Integer)

Each H01 synapse, neuron, and layer entity has an arbitrary amount of key-value properties which represent the object's metadata and attributes. Currently, each layer object has one attribute, each neuron object has 19 attributes, and each synapse object has seven attributes. Additionally, some attributes link to other entities with their own properties. For example, the synapse object has attributes, pre-synaptic site and post-synaptic partner, which contain sub-attributes, such as the associated neuron's id and class type. The document-oriented storage approach allows for H01 annotation attributes to be stored as arbitrary key-value pairs in which attributes can be easily added, queried, and indexed. This method served its primary purpose of demonstrating metadata standardization benefits, and we did not explore other database types.

### 4.3. Querying Data

**Figure 1:**
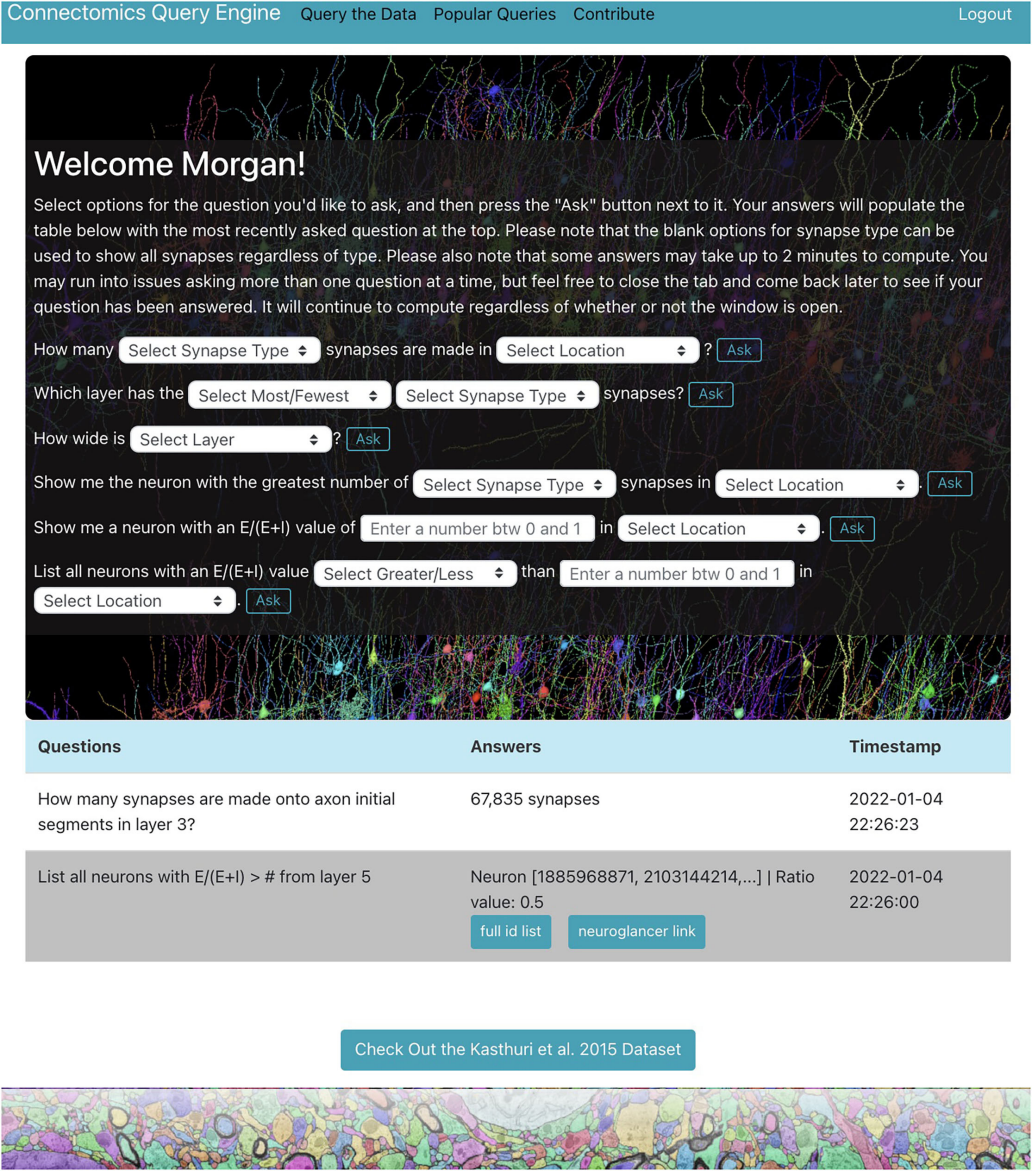
H01 personal query page. Users can ask questions about the H01 dataset using dropdown menus and text boxes. Some answers are well-suited for visualization. The app uses Neuroglancer to display neurons related to such answers.

We demonstrate a querying tool which performs standard queries relevant to a variety of connectomics research areas from the H01 dataset using the RAMON API. It is in the form of a web application with a user-friendly interface and provides a centralized location where users can easily explore the dataset individually through a personal query page (Figure 1), only accessible after the user is authenticated, as well as collaboratively through a “Popular Queries” page, accessible to all users.

The web application, located at metadata.bossdb.org, is also a Flask app which uses SQLite to store query and user information. It provides users with the ability to extract data via dropdown menus, enables visualization of cells and their synapses via Neuroglancer, supports reporting of potentially problematic annotations, compiles questions and answers from all users to generate a “Popular Queries” page, and even allows users to suggest new queries for integration into the app. At the moment, users can ask seven types of questions about the H01 dataset for a total of 119 questions shown below:

1. How many [Synapse Type] synapses are made in [Location]?
2. Which layer has the [Most/Fewest] [Synapse Type] synapses?
3. How wide is [Layer]?
4. What is the [Average/Total] length of neurons in [Location]?
5. Show me the neuron with the greatest number of [Synapse Type] synapses in [Location].
6. Show me a neuron with an $\frac{E}{E+I}$ value of [Value between 0 and 1] in [Location].
7. List all neurons with an $\frac{E}{E+I}$ value of [Greater/Less] than [Value between 0 and 1] in [Location].

where synapse types include all synapses, excitatory and inhibitory synapses, and those onto axon initial segments or dendrites, locations include the entire volume or any of the seven layers, and E and I are the number of incoming excitatory and inhibitory synapses, respectively.

This list of question types will continue to expand as functionality is added. We hope to continue to incorporate query types, especially those discussed in Section 3.

## 5. Discussion

Nanoscale connectomics is an exciting field that has the potential to answer a wide range of questions in neuroscience and the potential to impact exciting and diverse application areas. The number and size of EM and XRM datasets are growing, and with that growth comes an increased need for standardization of both imaging data and annotations. Work to standardize imaging data is underway, while standards to address annotation format and content variability are still emerging. As the field exists today, extracting relevant information about labeled entities requires both a significant computational background and a deep understanding of how that particular data is stored. Consequently, data access tools built upon annotation standards will increase accessibility and ease of access to these exciting datasets.

Standardization of metadata, while maintaining the flexible spirit embodied by an emerging field lays the groundwork for community-adopted standards which enable reproducible analysis as well as natural standards evolution over time. RAMON, RAMON API, and the H01 Community Discovery Portal demonstrate one example of how annotation standards and software tools can interact to support both collaborative and individual scientific discovery, but the possibilities are endless. The H01 Discovery Portal demonstrates how metadata standardization can push the field of connectomics toward solving potential applications by reducing redundant data processing code and encouraging data exploration and collaboration. All three of these tools have the potential to evolve to include additional queries, data sources, and annotation types.

Although it would be convenient to develop fully-automated pipelines to convert from lab-specific implementations to a common schema, due to the diversity in storage formats currently implemented, this will require future work. However, the process of understanding and translating important datasets has relatively low-resource requirements and can be simplified by focusing on the final, published data, which tends to be more standardized and common than intermediate products. The authors extended the portal to include a query page for Kasthuri et al. (2015) in addition to the H01 dataset. Though this data contained different entities, it existed in a tabular format similar to RAMON, which allowed for quick integration into the software stack.

The authors note, however, that the implementations of these tools may not be optimal as they were built primarily for standards demonstration purposes and thus serve as a proof of concept. For example, only MongoDB was considered for storing H01. In order to determine the best type of database to use for metadata storage, additional options such as DynamoDB, Google Cloud Firestore, Cassandra, and Azure Cosmos DB must be explored. Additionally, the web interface went through a small number of internal design cycles with particular emphasis on simple, clear, and intuitive querying. A more polished portal would necessitate an extensive design and feedback process. The authors hope to develop similar tools once standards are developed that allow for intuitive exploration of numerous datasets in a centralized location through expansion and integration of existing tools as well as development of new ones.

Further, we note that a top-down, universal specification of metadata standardsis unlikely to satisfy all stakeholders. For future work, we will, therefore, seek a data-driven approach leveraging existing published data (Hider et al., 2019) and explicit community input. Standardization is often a balance between flexibility and usability, and we believe a fruitful path forward is to concentrate initially on published products.

Given the current limited accessibility of connectomics data, the patterns in brain networks may remain hidden behind these complex data, and scientific discovery could be limited. We look forward to building on these initial tools and formats through community engagement and feedback.

## Data Availability Statement

Publicly available datasets were analyzed in this study. This data can be found here: https://h01-release.storage.googleapis.com/landing.html; https://bossdb.org/project/kasthuri2015.

## Author Contributions

MS and DM designed and developed the case study and validated the proposed standardization framework. EJ, BW, and WG-R proposed and defined the standards and perspectives outlined in this work, with input from the community. JL proposed the case study and provided neuroscience interpretation and design feedback. All authors contributed to the manuscript drafting and reviews and approved the submitted version of the manuscript.

## Funding

Research reported in this publication was also supported by the National Institute of Mental Health of the National Institutes of Health under Award Numbers R24MH114799, R24MH114785, U24NS109102, and R01MH126684.

## Author Disclaimer

The content is solely the responsibility of the authors and does not necessarily represent the official views of the National Institutes of Health.

## Conflict of Interest

The authors declare that the research was conducted in the absence of any commercial or financial relationships that could be construed as a potential conflict of interest.

## Publisher's Note

All claims expressed in this article are solely those of the authors and do not necessarily represent those of their affiliated organizations, or those of the publisher, the editors and the reviewers. Any product that may be evaluated in this article, or claim that may be made by its manufacturer, is not guaranteed or endorsed by the publisher.
